# A comparative investigation of the quality of radiographs produced by portable handheld and fixed x-ray units

**DOI:** 10.1590/0103-644020256319

**Published:** 2025-10-24

**Authors:** Débora Costa Ruiz, Matheus L. Oliveira, Rocharles Cavalcante Fontenele, Deborah Queiroz Freitas, Francisco Haiter-Neto

**Affiliations:** 1 Department of Oral Diagnosis, Piracicaba Dental School, University of Campinas, Piracicaba, SP, Brazil; 2 University Hospitals Leu" icountry="BE"> OM FS IMPATH Research Group, Department of Imaging and Pathology, Faculty of Medicine, KU Leuven and Oral and Maxillofacial Surgery, University Hospitals Leuven, Leuven, Belgium

**Keywords:** X-rays, Handheld, Dental digital radiography, Dental device

## Abstract

To evaluate the impact of handheld portable intraoral diagnostic X-ray equipment on radiographic quality and to compare their radiographs with radiographs obtained using fixed intraoral X-ray equipment. For brightness, noise, and uniformity, radiographs of an acrylic block were obtained with two complementary metal oxide semiconductor (CMOS) sensors (Digora Toto and Snapshot). Six radiographs for each sensor were obtained using the Eagle handheld portable X-ray equipment set at 60 kVp, 2.5 mA, and 0.45 s. Then, six radiographs were obtained with the Focus fixed X-ray equipment set at 60 kVp, 7 mA, and 0.16 s. Mean and standard deviation of the gray values were evaluated. For contrast, radiographs of an aluminum step-wedge were obtained using both sensors, X-ray equipment, and the aforementioned acquisition parameters. The percentage of contrast variation was evaluated. The comparison between the equipment was evaluated for its influence on radiographic quality using Student's t-test *(p < 0.05)*. The handheld portable X-ray equipment produced radiographs with higher brightness and lower contrast regardless of the CMOS sensor used *(p<0.0001)*. Noise levels were higher with the handheld portable X-ray equipment than with the fixed X-ray equipment using the Digora Toto system, and lower with the Snapshot system *(p < 0.05)*. No significant differences were found for uniformity. Therefore, radiographs obtained with a handheld portable intraoral diagnostic X-ray equipment have higher brightness and lower contrast compared to those obtained with fixed X-ray equipment. While the effect on noise levels varies depending on the digital system used, the uniformity remains unaffected by the type of X-ray equipment.

## Resumo

Objetivou-se avaliar o impacto de um equipamento de radiologia diagnóstica móvel na qualidade radiográfica e comparar suas radiografias com radiografias obtidas com um equipamento de raios X intraoral. Para avaliar brilho, ruído e uniformidade, radiografias de um bloco de acrílico foram obtidas com dois sensores de semicondutor de óxido metálico complementar (CMOS) (Digora Toto e Snapshot). Seis radiografias para cada sensor foram obtidas usando o equipamento móvel Eagle (60 kVp, 2,5 mA e 0,45s). Seis radiografias foram obtidas com o equipamento de raios X intraoral Focus (60 kVp, 7 mA e 0,16s). A média e o desvio padrão dos valores de cinza foram avaliados. Para o contraste, radiografias de uma escala de alumínio foram obtidas usando os sensores, aparelhos e parâmetros mencionados anteriormente. A porcentagem de variação de contraste foi avaliada. A comparação entre os equipamentos por meio de sua influência na qualidade radiográfica foi avaliada com o teste t de Student *(p<0,05)*. O equipamento móvel resultou em radiografias com maior brilho e menor contraste, independentemente do sensor usado *(p<0,0001)*. O ruído foi maior com o equipamento móvel do que com o equipamento fixo intraoral usando o sistema Digora Toto e menor com o sistema Snapshot *(p<0,05)*. Nenhuma diferença significativa foi encontrada para uniformidade. Portanto, radiografias obtidas com um equipamento de radiologia diagnóstica móvel têm maior brilho e menor contraste em comparação com um equipamento de raios X intraoral. Já o ruído varia de acordo com o sistema digital empregado e a uniformidade não é influenciada pelo tipo de equipamento.

**Figure:**
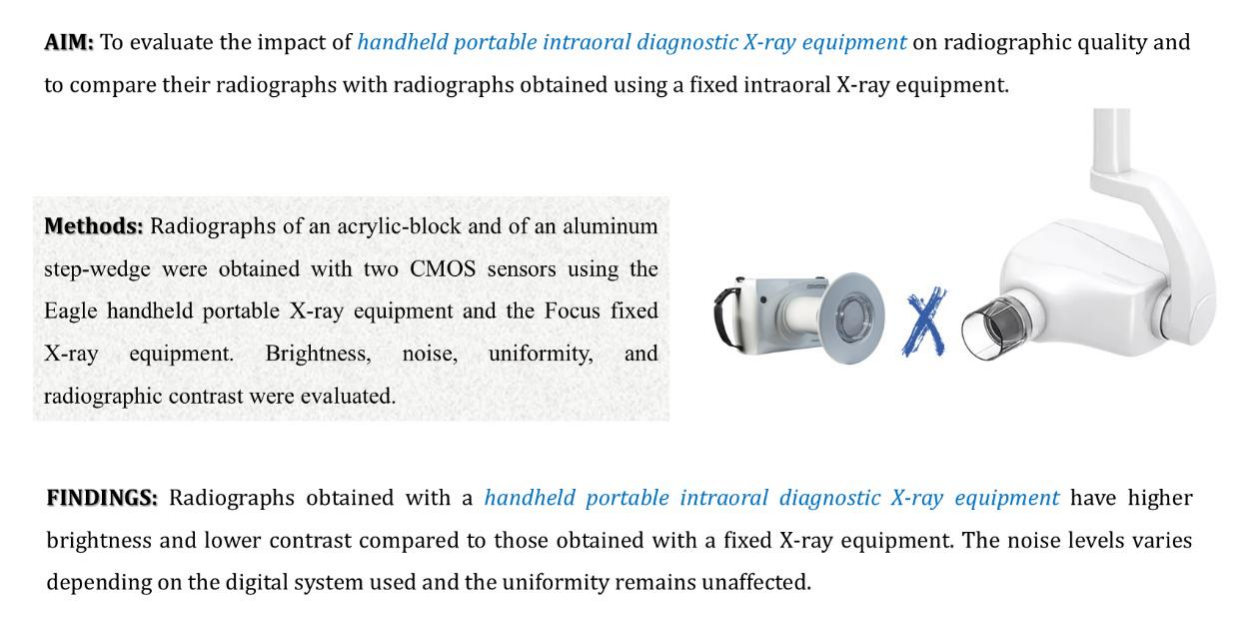

## Introduction

Initially developed in the early 90s to aid dental care for soldiers in military operations, handheld portable intraoral diagnostic X-ray equipment was an alternative to fixed intraoral X-ray equipment, facilitating intraoral radiographic acquisitions in the field^1^ ^,^ ^2^. Over the past decade, the use of handheld, portable intraoral diagnostic X-ray equipment has expanded in daily clinical practice, highlighting the need for studies that explore its characteristics, applicability, and the implications of its widespread use^1^ ^,^ ^2^ ^,^ ^3^.

A primary concern with this equipment is the dose of ionizing radiation delivered to both patients and operators. The consulted literature indicates that the biological risks associated with handheld portable intraoral diagnostic X-ray equipment are comparable to those of fixed intraoral X-ray equipment^4^. Additionally, studies evaluated factors such as aiming precision, potential distortion in intraoral radiographs, the relationship between battery levels and tube voltage, and the use of handheld portable intraoral diagnostic X-ray equipment in forensic dentistry ^5^ ^,^ ^6^ ^,^ ^7^ ^,^ ^8^. While the results are generally promising, a critical gap remains in the literature regarding the impact of handheld, portable intraoral diagnostic X-ray equipment on radiographic quality.

Objective studies enable the evaluation of radiographic quality, typically involving the analysis of gray values in radiographs to provide insights into brightness, noise, uniformity, and contrast ^9^ ^,^ ^10^ ^,^ ^11^ ^,^ ^12^. These evaluations are crucial not only for understanding the impact of using handheld portable intraoral diagnostic X-ray equipment but also for determining how they affect different types of digital systems. Once variations in gray values can potentially influence the accuracy of diagnostic tasks, our study aimed to evaluate the objective image quality of radiographs obtained with a handheld, portable intraoral diagnostic X-ray equipment using different complementary metal-oxide-semiconductor (CMOS) sensors and compare their quality to that of radiographs obtained with a fixed intraoral X-ray equipment.

## Material and methods

**Figure 1:**
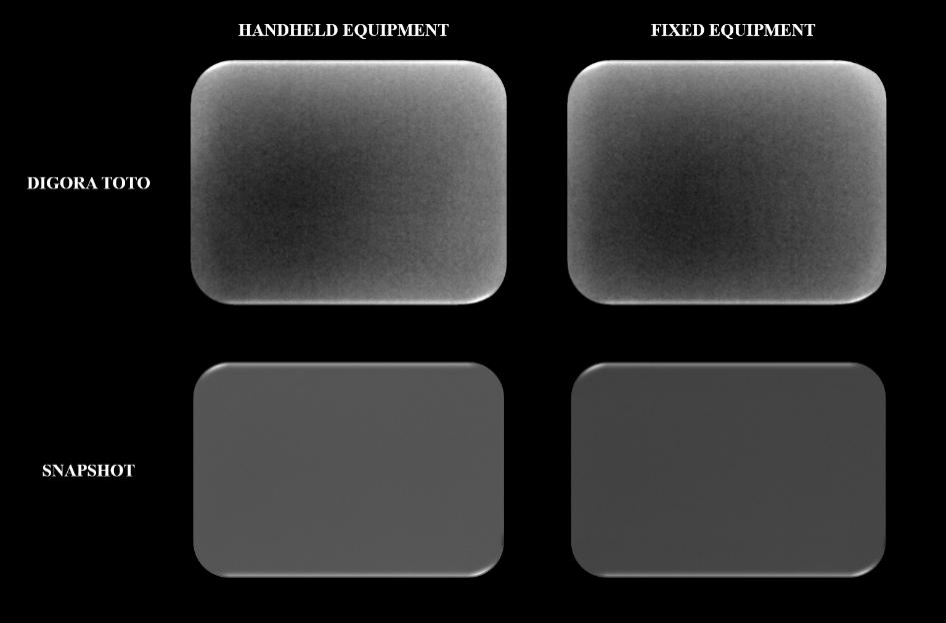
Radiographs of an acrylic block obtained with handheld portable X-ray and fixed intraoral X-ray equipment using the Digora Toto and Snapshot systems.

To evaluate brightness, noise, and uniformity, an acrylic block measuring 30 mm in height, 40 mm in length, and 30 mm in width was radiographed using a size 2 CMOS sensor from Digora Toto system and the Scanora software (Soredex, Tuusula, Finland). Six radiographs were obtained based on the paralleling technique with the Eagle X-ray handheld portable intraoral diagnostic X-ray equipment (Alliage, São Paulo, Brazil), set at 60 kVp, 2.5 mA, and an exposure time of 0.45 seconds. This process was repeated, with six more radiographs obtained using the same technique and exposure parameters for a size 1 CMOS sensor from the SnapShot system and the Cliniview software (Instrumentarium Imaging, Milwaukee, United States) (Figure 1). For all radiographic acquisitions, the handheld portable intraoral diagnostic X-ray equipment was mounted on a stable platform. This approach was adopted to eliminate potential variability caused by hand movement or positioning differences during manual operation, thereby ensuring that operator-related factors did not influence the quality of the radiographs.

Additional radiographs were obtained using a Focus fixed intraoral X-ray equipment (Instrumentarium, Tuusula, Finland) set at 60 kVp, 7 mA, and an exposure time of 0.16 seconds. Again, six radiographs were obtained for each of the two CMOS sensors tested, following the aforementioned methodology (Figure 1).

The exposure times selected for the X-ray equipment were chosen after two dentomaxillofacial radiologists with five years of experience conducted a pilot study that subjectively evaluated different exposure times and their effects on the radiographic quality. A time of 0.16 seconds was selected for the fixed intraoral X-ray equipment because it yielded radiographs with optimal brightness and contrast. Then, a time of 0.45 seconds was selected for the handheld portable intraoral diagnostic X-ray equipment, as the product of the milliampereage and time (mA × seconds of exposure to X-rays) was similar for both pieces of equipment (1.125 mAs for the handheld equipment and 1.12 mAs for the fixed equipment). 

**Figure 2:**
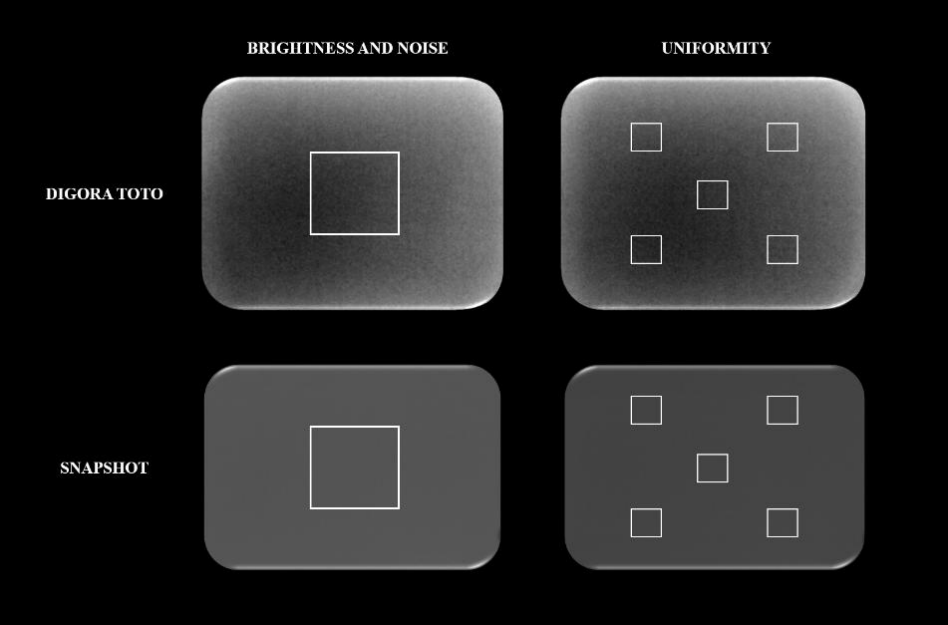
Radiographs with empty white squares representing the ROIs for brightness, noise, and uniformity evaluation. *ROI, region of interest*

All the resulting 24 radiographs (6 radiographs per unit × 2 equipment × 2 digital systems) were exported in 8-bit TIFF file format. Using the ImageJ software (National Institutes of Health, Maryland, United States), for brightness and noise evaluation, a square region of interest (ROI) was placed in the central region of the radiograph, covering 16% of its total area (Figure 2) ^9^ ^,^ ^10^. Mean gray values and standard deviation (SD) of the gray values were collected and used to evaluate brightness and noise, respectively ^9^ ^,^ ^10^.

To evaluate uniformity, an ROI measuring 4 × 4 mm was positioned in the center of the radiograph, followed by four ROIs of the same size, symmetrically distributed on the upper and lower sides of the radiograph. Then, the SDs of the gray values of the five ROIs were collected and averaged (Figure 2) ^9^ ^,^ ^10^. The macro function of the software was employed to standardize the location of the ROIs for all radiographs.

**Figure 3:**
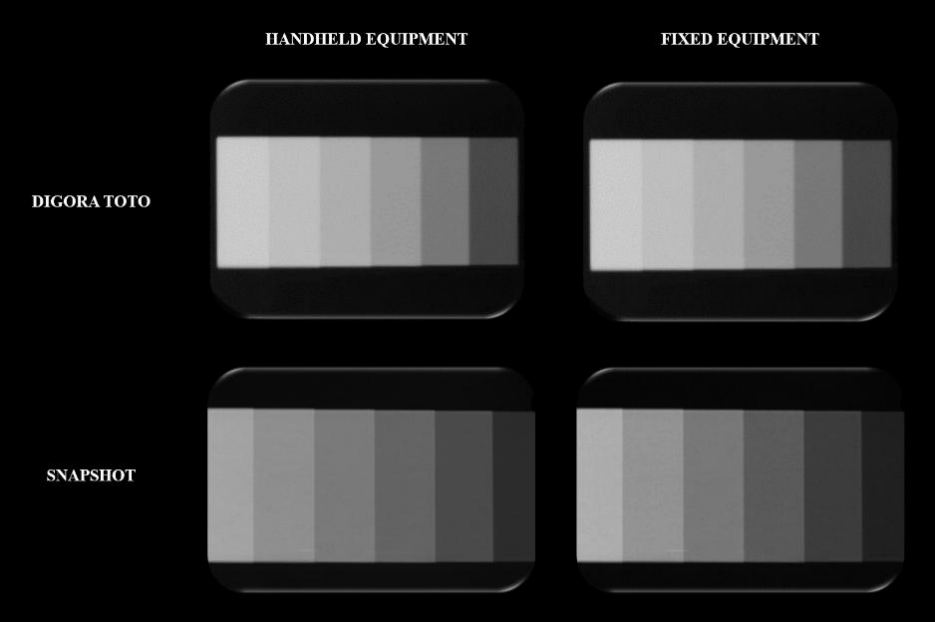
Radiographs of an aluminum step-wedge obtained with handheld portable X-ray and fixed intraoral X-ray equipment using the Digora Toto and Snapshot systems.

To evaluate the radiographic contrast, the same digital radiographic systems, acquisition parameters, and X-ray equipment previously described were used to obtain new radiographs. This time, an aluminum step-wedge with six steps of increasing thickness (2,4,6,8,10, and 12 mm) was placed horizontally and centered on the CMOS sensors for the acquisitions (Figure 3).

**Figure 4:**
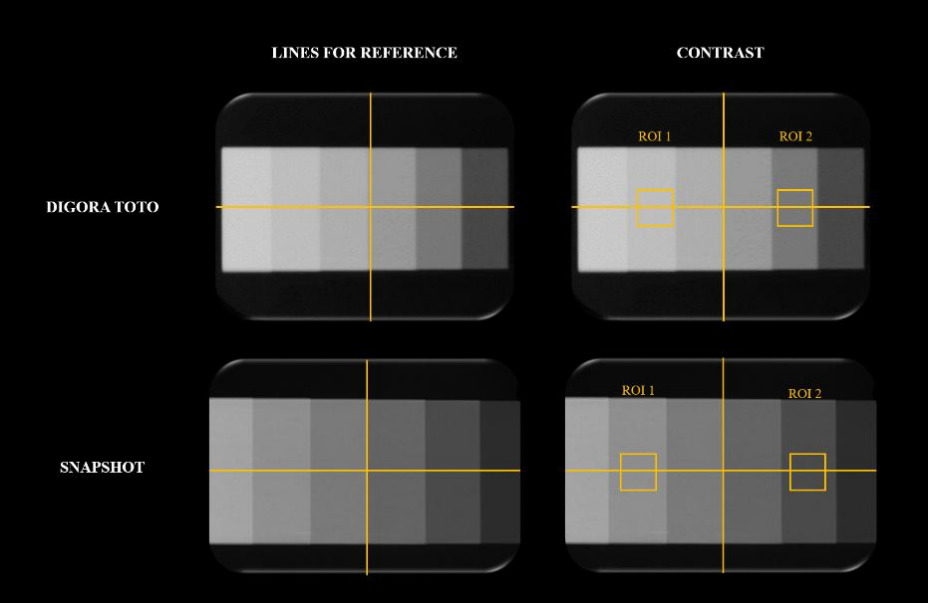
Radiographs with vertical and horizontal yellow lines as references were used to place ROIs 1 and 2 for contrast evaluation. *ROI, regionof interest*

All 24 resulting radiographs (6 radiographs per unit × 2 equipment × 2 digital systems) were exported and analyzed using ImageJ software. A vertical line was drawn in the center of the radiograph of the step-wedge. Then, a horizontal line was drawn to equally divide the radiograph of the step-wedge (Figure 4). Subsequently, one square ROI (4 × 4 mm) was positioned in the center of the aluminum step with 10 mm thickness (ROI 1), and another ROI (4 × 4 mm) was placed in the center of the aluminum step with 4 mm thickness (ROI 2) (Figure 4). The macro function was used to standardize the positions of the ROIs. The percentage of contrast variation was calculated according to the formula ^11^ ^,^ ^12^:

$$Percentage\;of\;contrast\;variation\;=\frac{\left(Mean\;of\;ROI\;1\;-Mean\;of\;ROI\;2\right)\times 100}{Mean\;of\;ROI\;1}$$

The values of brightness, noise, uniformity, and percentage of contrast variation were compared between the tested equipment using Student’s t-test. The significance level was set at 5% *(α = 0.05).* The power analysis for the test was 95% for all evaluated variables. The statistical analyses were performed using the SPSS 25.0 (SPSS Inc., Chicago, Illinois, USA) software.

## Results

For radiographs of the acrylic block, a significant difference in brightness and noise was observed, depending on the type of equipment and CMOS sensor used (p < 0.05), as shown in Table 1. Specifically, when the Digora Toto system was employed, the handheld, portable intraoral diagnostic X-ray equipment resulted in increased brightness and noise (p < 0.0001). Similarly, for radiographs obtained with the Snapshot system, brightness increased with the handheld portable intraoral diagnostic X-ray equipment (p<0.0001). Moreover, the noise was lower on radiographs obtained with the handheld portable intraoral diagnostic X-ray equipment compared to those obtained with the fixed intraoral X-ray equipment (p = 0.001). At the same time, uniformity values did not change significantly for the CMOS sensors tested (p > 0.05). Table 1 also shows the percentage of contrast variation based on radiographs of the aluminum step-wedge. According to the findings, for both digital systems, the contrast on radiographs obtained with the handheld portable intraoral diagnostic X-ray equipment decreased compared to radiographs obtained with the fixed intraoral X-ray equipment (p<0.0001).

**Table 1:** Mean (standard deviation) of the radiographic quality parameters according to the digital system and X-ray equipment

Digital System	X-ray Equipment	Brightness	Noise	Uniformity	Contrast
Digora Toto	Handheld Portable	65.41 (0.86)	14.48 (0.24)	9.48 (0.23)	31.85 (0.04)
	Fixed	57.66 (1.04)	13.10 (0.27)	9.53 (0.12)	32.99 (0.03)
*p value*		<0.0001	<0.0001	0.661	<0.0001
Snapshot	Handheld Portable	85.38 (0.19)	1.05 (0.02)	1.00 (0.08)	42.81 (0.18)
	Fixed	68.95 (0.27)	1.12 (0.01)	1.03 (0.00)	47.69 (0.02)
*p value*		<0.0001	0.001	0.445	<0.0001

## Discussion

Although the consulted literature revealed a substantial number of studies focused on radioprotection and forensic applications of handheld, portable intraoral diagnostic X-ray equipment, a notable scarcity of research remains evaluating the quality of radiographs produced by this equipment ^4^ ^,^ ^6^ ^,^ ^8^. This represents a significant gap, particularly given the growing use of portable intraoral diagnostic X-ray equipment in clinical settings. Radiographic quality is a critical factor for accurate diagnosis, treatment planning, and patient care across a wide range of dental conditions. Since differences in image brightness, noise, uniformity, or contrast can impact the clinician's ability to interpret radiographic findings, studies that objectively assess the quality of radiographs are essential to ensure that their convenience and portability do not compromise diagnostic reliability. For example, excessive brightness and reduced contrast can hinder the differentiation between adjacent tissues or structures, thereby complicating diagnostic interpretation. Similarly, poor uniformity or increased image noise may degrade sharpness and compromise visual clarity, further limiting diagnostic confidence. In this context, analyses based on the gray values of radiographs serve as a first step toward understanding the potential clinical implications of variations in radiographic quality. According to the present study, a handheld, portable intraoral diagnostic X-ray equipment produces radiographs with higher brightness and lower contrast compared to fixed intraoral X-ray equipment, regardless of the digital system used. For the noise, this interpretation differed: noise levels were higher when the handheld portable intraoral diagnostic X-ray equipment was used with the Digora Toto system, but lower when used with the Snapshot system.

Radiographic quality can be investigated through different methodological approaches. Nitschke et al. evaluated the quality of radiographs obtained with handheld, portable intraoral diagnostic X-ray equipment - manually held by clinicians - by assessing the geometric distortion of anatomical structures ^6^. Their study employed a subjective evaluation method in which two examiners analyzed key anatomical landmarks (such as the periodontal ligament space, lamina dura, periradicular bone, alveolar crest, pulp chamber, root canals, and dental crowns) to detect potential distortions when comparing radiographs obtained with handheld versus fixed equipment ^6^. Based on their findings, no meaningful differences were reported between the two types of equipment ^6^. In contrast, the present study adopted an objective assessment approach based on the analysis of gray values. The differences in findings between the two studies may be attributed to the nature of the evaluation methods used. While Nitschke et al. relied on examiner perception to detect geometric distortions, the present study employed quantitative, software-based measurements. Despite these methodological differences, both studies make meaningful contributions to the broader understanding of the performance of handheld, portable intraoral diagnostic X-ray equipment. Each study targets a distinct aspect of radiographic quality, and together, these complementary perspectives provide valuable information regarding the suitability of handheld, portable intraoral diagnostic X-ray equipment.

Curiously, a previous study evaluated the quality of radiographs obtained with a handheld portable intraoral diagnostic X-ray equipment combined with a photostimulable phosphor (PSP) plate receptor ^13^. The study found that these radiographs exhibited higher brightness and lower contrast. Notably, although the current study used CMOS sensors instead of a PSP receptor, similar alterations in brightness and contrast were observed. This convergence of results suggests that handheld, portable intraoral diagnostic X-ray equipment may have certain inherent technical features that could contribute to changes in brightness and contrast. However, the type of digital system applied also appears to affect the quality of radiographs, as noise levels were higher for radiographs obtained with the Digora Toto system and the handheld portable intraoral diagnostic X-ray equipment compared to those obtained with the Snapshot system and the same equipment. This result suggests that variations in CMOS sensor sensitivity between the two systems can influence the signal-to-noise ratio. Each system may employ distinct image processing algorithms, which can influence the gray values of radiographs ^14^ ^,^ ^15^. Additionally, differences in their components, such as scintillator materials, analog-to-digital conversion mechanisms, and detector electronics, can also influence noise characteristics ^16^.

Moreover, an important aspect of the noise must be discussed. Despite the statistically significant noise changes observed, the numerical difference was minimal compared to the numerical differences for brightness and contrast. In this case, noise changes could be imperceptible to the human eye; once these differences became statistically significant, it was primarily due to the low standard deviation. These findings align with the concept that human vision has a limited capacity to detect subtle grayscale variations compared to the precision of software-based analyses, such as those performed in the present study ^17^. This idea is supported by previous research on the prolonged use of PSP plate receptors, which reported an increase in radiographic brightness over time ^9^. However, this increase did not compromise the radiographic diagnosis of caries lesions, reinforcing the idea that quantitative changes may not always translate into clinically perceptible differences ^9^ ^,^ ^18^. Therefore, although noise values on radiographs obtained with a handheld portable intraoral diagnostic X-ray equipment were statistically different, the authors believe that these values would not impair the diagnosis in a real clinical scenario.

The existing literature presents previous studies that assess the influence of using handheld portable intraoral diagnostic X-ray equipment in a clinical scenario for the radiographic diagnosis of caries lesions ^15^ ^,^ ^19^. According to previous studies, the radiographic diagnosis of proximal caries lesions was not compromised by handheld, portable intraoral diagnostic X-ray equipment ^15^ ^,^ ^19^. These results may appear counterintuitive, as the present study observed differences in gray values of radiographs. However, it is essential to note that statistical differences in radiographic quality parameters do not necessarily translate into clinically significant diagnostic differences ^9^ ^,^ ^20^. Moreover, it is essential to recognize that studies designed to evaluate radiographic quality objectively differ significantly from those focused on diagnostic performance. Objective studies aim to evaluate specific technical parameters. In contrast, diagnostic studies involve a more complex set of variables that extend beyond image quality alone. The diagnostic accuracy can be influenced by factors such as the size, location, and visibility of the lesion, examiner's level of experience, and post-processing adjustments of radiographs; this last one can promote changes on the grayscale of radiographs regardless of any changes inherently introduced using handheld portable intraoral diagnostic X-ray equipment ^14^ ^,^ ^21^ ^,^ ^22^.

However, it is essential to note that previous diagnostic assessments utilizing handheld, portable intraoral diagnostic X-ray equipment have primarily focused on the detection of caries lesions ^15^ ^,^ ^19^. While valuable, this singular diagnostic context limits the generalizability of the findings. Different dental conditions, such as root resorptions, root fractures, or periodontal bone loss, exhibit unique radiographic features and diagnostic challenges. These conditions involve complex anatomical structures that, radiographically, exhibit varying densities, shapes, and contrasts. As a result, the performance of handheld portable intraoral diagnostic X-ray equipment may vary among the diagnostic tasks assessed. Hence, the diagnostic performance of handheld, portable intraoral diagnostic X-ray equipment cannot be fully understood based on caries detection alone; more diagnostic studies are needed to comprehend the clinical reliability of this type of equipment.

While the contrast was assessed through radiographs of an aluminum step-wedge, following a well-established methodology in the literature ^9^ ^,^ ^11^ ^,^ ^12^, brightness, noise, and uniformity were assessed through radiographs of an acrylic block positioned between the X-ray equipment and the CMOS sensors. The homogeneity of the acrylic block served as a fundamental prerequisite, minimizing the high variability typically associated with anatomical structures and ensuring uniform interaction with the X-ray beam, thereby resulting in a homogeneous radiograph. This condition is crucial for accurately assessing image noise and uniformity. For instance, the human mandible exhibits considerable inter-individual differences in shape, density, and composition, which could introduce subjectivity to the results. Such variability would detract from the primary aim of this research, which was to objectively evaluate how the use of a handheld portable intraoral diagnostic X-ray equipment influences the gray values in radiographic images. Moreover, the use of an acrylic block is consistent with the methodology adopted in previous studies that analyzed gray values, ensuring a standardized and reproducible approach to radiographic quality assessment ^9^ ^,^ ^10^ ^,^ ^12^ ^,^ ^13^ ^,^ ^23^.

Positioning the handheld portable intraoral diagnostic X-ray equipment on a stable platform was a deliberate methodological choice to standardize radiographic acquisition and eliminate potential sources of variability. Handheld equipment typically weighs between 2.5 and 5 kilograms ^1^ and requires the operator to manually hold it throughout multiple X-ray exposures, which can introduce inconsistencies due to hand movement or fatigue. These factors could compromise radiographic quality by affecting the stability and precision of the X-ray beam alignment. By fixing the equipment in place, the study ensured consistent exposure geometry across all radiographs; thereby, the differences observed in the radiographic quality can be more confidently attributed to the characteristics and performance of the equipment itself, rather than to inconsistencies introduced by manual handling during exposure.

All radiographs were obtained using the handheld, portable intraoral diagnostic X-ray equipment that was fully charged. This approach was based on a previous study that found a reduction in tube voltage as the battery charge decreased, which could affect radiographic quality ^7^. Based on this evidence, it is reasonable to suppose that a decline in battery level could result in diminished power output, potentially affecting the quality of radiographs. In the present study, we used fully charged equipment to ensure standardization and eliminate battery-related variability. However, in routine clinical practice, maintaining the equipment at full charge may not always be possible, particularly in small clinics or mobile care settings. This raises concerns about the feasibility of consistently achieving high-quality radiographs without frequent recharging. Therefore, the present study highlights the need for future studies to evaluate this topic. Such investigations would be essential for developing evidence-based recommendations on battery management protocols.

Future research on the influence of battery levels in handheld portable intraoral diagnostic X-ray equipment represents just one of several important recommendations stemming from the present study. While the objective analysis provided valuable insights into how this type of equipment may affect radiographic gray values, further investigation is essential to understand its impact in real-world clinical settings fully. Therefore, the authors encourage future studies to explore the effects of varying exposure parameters. Specifically, it would be valuable to explore whether extending the exposure time has a similar impact on radiographic quality when using handheld X-ray equipment compared to fixed intraoral X-ray equipment. Additionally, future studies should investigate whether software-based adjustments of brightness and contrast can effectively compensate for the limitations in radiographic quality associated with the use of handheld portable intraoral diagnostic X-ray equipment, once it can be hypothesized that increasing the brightness and reducing the contrast of radiographs, which aligns with the inherent characteristics observed in handheld-obtained radiographs, may further compromise the visibility of diagnostic features. In contrast, adjusting these parameters in the opposite direction -by reducing brightness and enhancing contrast -could potentially improve radiographic quality, making them more comparable to those obtained with fixed intraoral X-ray equipment. This suggests that the clinician's choice of post-processing strategy could either mitigate or exacerbate the inherent radiographic quality differences between handheld and fixed equipment, highlighting the need for future studies to explore this interaction. Finally, given that handheld portable diagnostic X-ray equipment is heavy, it is essential to investigate whether the act of manually holding the equipment during radiographic acquisition affects radiographic quality, as the physical demands of supporting this device may lead to unintentional movements or deviations.

Based on objective analyses, radiographs obtained with handheld portable intraoral diagnostic X-ray equipment have higher brightness and lower contrast compared to radiographs obtained with fixed intraoral X-ray equipment, regardless of the digital system used. The effect of noise may vary depending on the CMOS sensor used. Additionally, the use of handheld, portable intraoral diagnostic X-ray equipment does not affect the uniformity values.
